# Historical trauma as a contributor to postpartum depression among Indigenous mothers

**DOI:** 10.3389/fpsyt.2026.1638586

**Published:** 2026-07-27

**Authors:** December Maxwell, Ric Thomas Munoz

**Affiliations:** Anne and Henry Zarrow School of Social Work, The University of Oklahoma, Norman, OK, United States

**Keywords:** historical trauma and historical oppression, Indigenous people, maternal health, maternal mental health, postpartum depression (PPD)

## Abstract

**Introduction:**

Postpartum depression (PPD) is a significant public health concern. Moreover, research suggests that American Indian/Alaskan Native (AI/AN) mothers experience postpartum depression at higher rates than the general population (Heck, 2021; Ko et al., 2017). As a result, understanding the potential causes of higher PPD among AI/AN mothers is helpful to the development of better interventions to reduce PPD among AI/AN mothers. A potential cause of higher PPD symptoms among AI/AN mothers is historical trauma (HT). HT refers to the cumulative psychological wounding experienced across generations due to systemic oppression, colonization, slavery, and other traumatic events experienced by a group of individuals.

**Methods:**

To test the theorized relationships between HT and PPD symptoms, we conducted an online survey of adult women who both identified as Indigenous and who give birth in the past five years (N = 56). The survey consisted of the psychometrically suitable measures of the Historical Loss Scale (HLS: Whitbeck at al, 2004) and the Edinburgh Postnatal Depression Scale (EPDS; Cox et al., 1987). .

**Results:**

The results indicated that a model of HLS scores as a predictor of PPD symptoms over and above the controls of income, mental health diagnosis, and the total number of children of the birth mother fit the data well (*χ*^2^ = 13.60; *df* = 10; *p* = .192; RMSEA = .081 [90% CI: .000, .178]; CFI = .949). Moreover, of all the predictors, the dimension of the HLS measuring mothers’ endorsement of the presence of oppressive governmental and institutional policies toward AI/AN was the strongest predictor of greater PPD (*β* = .28, *p* < .05). A Bollen Stine bootstrap test was also used to confirm the stability of the model. The Bollen Stine operates by comparing the theorized model to a model that perfectly fits the data. The results of the Bollen Stine test indicated that the theorized model was not significantly different than a model of perfect fit. .

**Discussion:**

Such results suggest that a potential explanation of higher PPD symptoms among AI/AN mothers is the effects of HT. Future interventions to reduce PPD symptoms among AI/AN mothers may benefit from an additional focus on treating HT.

## Introduction

Historical trauma (HT), also known as collective trauma, refers to the cumulative emotional and psychological wounding experienced across generations due to systemic oppression, colonization, slavery, and other collective traumatic events, has profound and enduring effects on individual and community well-being as a result of traumatic events or experiences that occurred in the past often spanning across generations (1). This concept is particularly relevant in understanding the mental health challenges faced by populations subjected to such adversities. Postpartum depression (PPD), a significant mental health condition characterized by persistent sadness, anxiety, and fatigue following childbirth (DSM-5-TRR, 2022), affects approximately between 7% to 63% of women globally (2). Moreover, research suggests that AI/AN mothers experience postpartum depression at higher rates than the general population (3, 4). While PPD is widely recognized as a pressing public health issue, its relationship with the intergenerational effects of HT remains underexplored in empirical literature. To develop interventions to help reduce PPD among this subpopulation, a better understanding of the potential causes of the higher rate of PPD is needed. For the current study, we sought to evaluate perceptions of HT as a potential antecedent of PPD among a sample of AI/AN mothers.

This study aims to statistically analyze the relationship between HT, as measured by the historical loss scale (HLS; 5), and PPD symptoms, among a sample of women who identified as indigenous. Grounded in the theoretical framework Historical Trauma Framework (including epigenetic models of trauma transmission) this study provides empirical data on how historical trauma influences PPD. Historical trauma provides a framework to understand unique risk factors for PPD in these populations, beyond typical biological or individual-level causes. Studying historical trauma also integrates sociocultural and intergenerational factors into models of maternal mental health, providing a more comprehensive understanding of PPD.

Populations historically affected by systemic oppression and collective trauma, such as Indigenous peoples and descendants of enslaved individuals, often experience higher rates of mental health challenges, including PPD (6). As such, understanding historical trauma is crucial for developing effective interventions and support systems for affected communities, as well as for promoting broader social understanding and healing. Based on the current literature and previous findings, we believe there will be a statistically significant positive correlation between experiencing historical trauma and developing postpartum depression.

## Supporting literature

### Postpartum depression

Perinatal mood and anxiety disorders (PMADs) address the spectrum of perinatal mental health challenges, Including depression, anxiety, posttraumatic stress disorder (PTSD), obsessive compulsive disorder, and postpartum psychosis during and after pregnancy (e.g., 7). Postpartum depression (PPD) is a major depressive disorder clinically defined by the Diagnostic and Statistical Manual of Mental Disorders (DSM) as depressive symptoms following birth of a child including apathy; lack of interest in new baby; anxiety about baby; anxiety about a lack of bonding; feelings of being a bad mother; feeling hopeless, worthless, and sad; and a fear of harming oneself or baby (American Psychological Association, 2022). Anywhere between 7% to 63% of mothers will experience PPD globally (2).

#### Prevalence and risk factors

Risk factors for postpartum depression (PPD) include a combination of biological, psychological, and social factors. Hormonal changes after childbirth, particularly fluctuations in estrogen and progesterone, can significantly affect mood regulation (8). Psychological factors such as a history of depression, anxiety, or other mental health disorders increase vulnerability, as do stressful life events like financial difficulties or relationship challenges (8, 9). Social factors, including lack of support from partners, family, or friends, can exacerbate feelings of isolation (8). Additionally, sleep deprivation (10) and physical exhaustion from caring for a newborn contribute to emotional distress (9). Certain populations may face heightened risk due to cultural stigma, systemic inequities, or historical trauma, which intersect with these factors to magnify the likelihood of PPD.

#### Impact of PPD

Postpartum depression (PPD) has significant and far-reaching consequences for maternal health, infant development, and family dynamics (11). Untreated PPD can lead to persistent depression in mothers, increasing their risk of future depressive episodes and potentially impacting their physical health (11). For infants, maternal PPD can interfere with bonding and attachment (9), potentially leading to delays in social, emotional, cognitive, and physical development. Children of mothers with long-term PPD are more likely to experience behavioral problems, academic challenges, and mental health issues later in life. PPD can also strain family relationships, affecting the mother’s ability to care for her child, potentially leading to early discontinuation of breastfeeding, and increasing the risk of depression in partners (Slomian et al., 2023). The impact of PPD extends beyond the immediate postpartum period, potentially creating an environment that hinders both maternal well-being and optimal child development (Slomian et al., 2023).

### Historical trauma: conceptual framework

The concept of Historical Trauma was first developed by Maria Yellow Horse Brave Heart while working with Lakota communities in the 1980s. This framework consists of three primary elements: a collective trauma experienced by a group, the sharing of this trauma by members of the group, and the spanning of this trauma across generations (12). The model posits that contemporary members of affected groups may experience trauma-related symptoms without having directly experienced the original traumatic events (12). Historical trauma is understood as a narrative representation that connects past group-experienced traumatic events to present-day experiences and contexts, including the contemporary health of a group or community (12). This framework incorporates aspects of psychosocial theory, political/economic theory, and social/ecological systems theory to provide a comprehensive understanding of how historical events can impact health disparities and disease prevalence in certain populations (13). The historical trauma framework also recognizes the importance of cultural resilience and adaptive responses, such as increased family and tribal cohesiveness, which can help communities cope with the effects of historical trauma (12).

#### Mechanisms of transmission

Historical trauma can manifest in numerous ways, including substance abuse, depression and anxiety, low self-esteem, anger and violence, difficulty expressing emotions, and suicidal thoughts (14, 15). The effects of historical trauma can be passed down through generations, influencing behaviors and mental health outcomes in descendants who didn’t directly experience the original traumatic events (1). Furthermore, historical trauma is also a “reaction to violent experience(s)” (16, p.5) and can be stored within memories, passed through stories and interpersonal violence, and can influence chronic stress within a community (17). Correspondingly, navigating unresolved grief is a process that is reactionary to continued cultural devastation, including the destruction of burial grounds and customs (1). In addition, historical trauma can affect entire societies, potentially shifting cultural norms and collective behaviors.

Given that historical trauma significantly influences the social determinants of health for communities affected by systemic oppression and collective traumatic events, it follows that historical trauma might also serve as a driver of the greater PPD among AI/AN mothers. The legacies of forced displacement, cultural erasure, and systemic discrimination disrupt traditional ways of life and erode the foundations of physical, mental, and social well-being (14, 15). These histories often lead to intergenerational cycles of poverty, reduced access to education, healthcare, and economic opportunities, and entrenched inequities that persist across generations (14, 15). Such barriers can result in higher rates of chronic illnesses and potentially mental health challenges such as PPD.

#### Populations impacted and trauma through policy

Based on what is known about the nature of historical trauma, our interests in the current study were to determine the unique contribution, if any, of HT associated with oppressive public policy on individual differences in AI/AN mothers’ PPD. We theorized a link between the two variables because others have noted that policies worsening HT often leads to psychological distress (15). Examples of oppressive public policies relating to AI/AN people include broken government to government treaties, relocation programs, and poor treatment by government officials (5). Generational exposure to such policies may increase vulnerability to stressors, such as food insecurity and unsafe living conditions. In fact, exposure to such oppressive public policy may be a mechanism by which HT perpetuates adverse social determinants of health, creating enduring inequities that hinder the well-being and resilience of affected communities (18). This link may also explain, in part, the causes of higher rates of PPD among AI/AN mothers.

### Intersection of historical trauma and PPD: pathways to PPD

**Figure 1:**
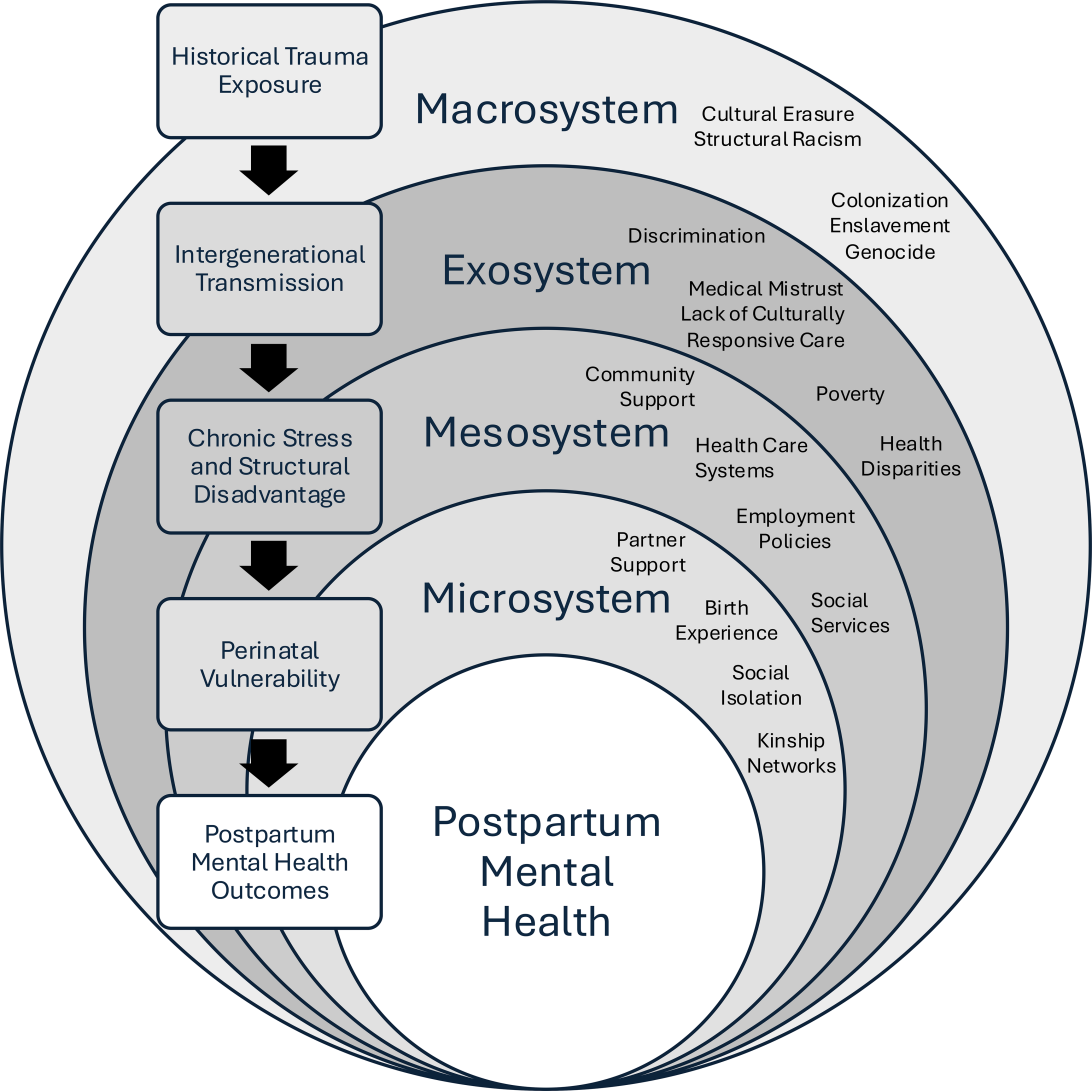
Conceptual model of historical trauma and postpartum mental health.

American Indian/Native American (AI/NA) mothers have the highest prevalence of PPD out of any other group in the United States; an estimated 17.5% of AI/NA mothers experience PPD (4). The prevalence of PPD within populations of descendants of enslaved people have not been specifically studied, however, research indicates that Black mothers have a higher prevalence of PPD than most ethnic and racial groups in the United States (19). Similarly, no direct research investigates the prevalence of PPD among offspring of Holocaust survivors or their descendants, however, research indicates descendants of Holocaust survivors are at a higher risk of anxiety and stress disorders due to familial trauma imparting impacts on cortisol responses (20). There are multiple factors that contribute to these higher rates which may be related to historical trauma and exacerbate PPD risk factors (see **Figure 1**), such as physical and mental health disparities, disparate poverty, cultural erasure, and communal isolation, which can be directly tied to a continued perpetuation of oppression and a result of HT (17b).

### Gaps in research

Qualitative research exists that examines the ways HT contributes to the exacerbation of PPD for some Indigenous women (21). However, the statistical link between HT and PPD has not been rigorously examined, leaving critical gaps in understanding how collective historical experiences manifest in maternal mental health. Addressing this gap is essential for designing effective, culturally responsive interventions to support women affected by both historical and postpartum challenges. To address this gap, this study asks the research question “Is there a positive correlation between experiencing HT and developing PPD?”. Based on existing theory, we hypothesized the data would indeed reflect a positive correlation between the two variables. Moreover, in a theory-based regression model, we specified HT as the independent variable and PPD as the dependent variable. Specifying *a prior* a directional theory of variable relationships is considered a best practice when statistically evaluating variable relationships using regression models and cross-sectional data (22). The specified model for the current study is based on the as theory that HT experienced over the lifespan is an antecedent of PPD symptoms experienced postpartum.

## Materials and methods

### Data collection

This study involved a sample of women who were at least 18 years old, had given birth within the past 5 years, who could read English, who self-identified as Indigenous. The cross-sectional, one-time study consisted of a nationwide survey using Qualtrics recruitment and data collection services. A Qualtrics panel is a recruitment and data collection service in which Qualtrics (an experience management platform that provides tools for designing surveys; collecting and analyzing data; and managing customer, employee, product, and brand experiences) sources participants that match study inclusion criteria (in this case women who had given birth in the past five years, were over 18 years old, resided in the U.S. and self-identified as Indigenous) and delivers their survey responses directly through the Qualtrics platform. Participants were recruited from various sources, including website intercept recruitment, member referrals, targeted email lists, gaming sites, customer loyalty web portals, permission-based networks, and social media, etc. Participants were awarded compensation in the form of loyalty points (per their preference) and points varied based on their use of the survey software and completion of survey. Participants were excluded if they were not 18 years old, did not live in the United States, or had not given birth in the past 5 years. The survey included demographics, the Historical Loss Scale (HLS; 5) and the Edinburgh Postnatal Depression Scale (EPDS; 23). Participation in the current study was voluntary, with IRB approval obtained from the university with which the authors are affiliated.

### Indigenous data sovereignty

To combat the negative effects of colonial research, the principle of tribal data sovereignty has emerged as to recognize the right of Indigenous people and tribes across the globe to govern the collection, ownership, and application of their data collected as part of research studies (24). Recommendations to promote data sovereignty include partnering with tribal governments in the management of tribal data.

To support protection of Indigenous interests and uphold principles of Tribal data sovereignty, the research team engaged in consultation with Tribal partners during study development. Although this national sample was not collected in partnership with a single Tribal Nation, two Tribal governments with whom members of the research team have existing, ongoing relationships were consulted regarding survey design, cultural appropriateness of question framing, and potential risks related to stigmatization. Additionally, these quantitative inquiries were informed by prior qualitative dissertation research conducted with the United Keetoowah Band, in which community members expressed interest in further investigation of historical trauma as a contributor to postpartum mental health. Collectively, these efforts were undertaken to ensure that the study addressed community-identified priorities, minimized potential harms, and aligned with emerging best practices in Indigenous research ethics, including respect for Tribal sovereignty and responsible representation of Indigenous data.

### Participants

All participants within the study identified as Indigenous mothers with an average age of 34.29 years (SD = 8.5). Additional demographics of the sample are reflected in **Table 1**.

**Table 1:** Sample demographics (N = 56).

Demographic	Raw total	% of Total (rounded)
Income		
0-19,000	9	16
20,000-45,000	12	21
46,000-75,000	7	13
76,000-100,000	14	25
101,000-150,000	11	20
Above 150,000	3	5
Mental health diagnosis		
Yes	25	45
No	31	55
Number of children		
1	20	37
2	21	39
3	8	15
4	3	6
5	2	4
Education		
Some high school	2	37
Completed high school	13	39
GED	2	15
Some college	8	6
Associates degree	4	4
Bachelor’s degree	19	37
Master's Degree	5	39
Doctoral Degree	1	15
Marital status		
Single	16	29
Married	29	52
Divorced	4	7
Widowed	2	4
Has partner	5	9

### Measures

#### Historical loss scale

Individual differences in participants’ perceptions of history loss were captured by the Historical Loss Scale (HLS; 5). The HLS asks participants to rate the frequency (1 = several times a day to 6 = never) with which they perceive various types of historical losses. The scale consists of 12 items, all reversed-scored, with a total score ranging from 12–72. Higher scores indicate greater perceptions of historical loss. The HLS has demonstrated good internal reliability and validity (5).

It should be noted that while initial research demonstrated that the HLS was adequately explained by a single latent factor (5), Whitbeck and colleagues (25) have since proposed that the HLS produces a two-factor model. Within a sample of adolescents, the two factor model of the HLS provided good fit (5).

Within the two-factor model of the HLS, factor one includes items that measure perceptions of oppressive government and institutional policies and practices, including loss of land, loss of family ties because of boarding schools, loss due to government relocation, broken treaties, and poor treatment by government officials. Factor two focuses on personal and cultural losses, including loss of language and spiritual ways, and loss of people to early death and through the effects of alcoholism.

For the current study, we modeled the HLS as the two-factor measure described above. The two factors of the HLS included factor 1, measuring perceptions of oppressive government and institutional policies, and factor 2, measuring personal and cultural losses. We sought to test whether the two-factor model produced good fit within a sample of Indigenous women. If the two-factor model of the HLS produced good fit, this would allow us to isolate what unique contributions to PPD symptoms were made by the experience of oppressive governmental and institutional policies relative to personal and cultural losses (5). We hypothesized that oppressive government policies would provide unique contributions to PPD relative to the contributions of personal and cultural losses.

#### Edinburgh Postnatal Depression Scale

The Edinburgh Postnatal Depression Scale (EPDS) is the most widely used screening tool designed to identify symptoms of postpartum depression in new mothers. Developed in 1987, the EPDS consists of 10 self-reported questions that assess the emotional well-being of individuals during the postpartum period, focusing on mood, anxiety, and depressive symptoms (23). Each item is scored on a scale of 0 to 3, with higher scores indicating a greater endorsement of PPD symptoms. The tool is validated for use across diverse populations and has been translated into multiple languages, making it an essential resource for early detection and intervention in postpartum health care.

#### Covariates

In testing the model of HLS scores as predictors of PPD symptoms, our analyses controlled for three covariates: (1) mental health diagnosis (coded as 0 if a participant reported no mental health diagnoses, and 1 reported she had a diagnosis); (2) income level, with higher scores indicating greater income; (3) the total number of children reported by participants.

The 3 variables in question were used as covariates because existing research suggests that mental health conditions (26), income level (27) and the number of children of a mother (28) are all associated with PPD. Employing covariates in a regression model increases the power of the model (29) to detect significant correlations between a selected independent variable, in this case HLS scores, and a dependent variable, in this case, PPD symptoms.

### Data analysis

Covariance based structural equation modeling (CB-SEM) was used to test a model of historical trauma as a driver of postpartum depression among Indigenous mothers. We used the SPSS Amos add-on (30) to perform all calculations. All such fit calculations were performed using maximum likelihood (ML) estimations.

We employed multiple fit tests to evaluate the proposed theoretical model. First, a χ2 analysis was used with a threshold of *p* >.05 indicating acceptable fit (31). We utilized additional fit tests that included the Comparative Fit Index (CFI) with a threshold of ≥.90 as an indication of acceptable fit (Bentler, 1992; Hu & Bentler, 2009). We also employed the Root Mean Square Error of Approximation (RMSEA), with a threshold of ≤.10 indicating acceptable fit (32).

### Nested models

To further strengthen the quality of our testing, we compared the theorized model to an alternative model. Our comparison involved comparing nested models. In the context of CB-SEM, nested models occur when a model contains freely estimated parameters that are a subset of another model (Bollen, 1989). To determine which model better explains the data, when additional paths are added to a model, the resulting Δ*χ*^2^ is tested for statistical significance. If the Δ*χ*^2^ created by the addition of a path is statistically significant, the path is retained in the final model (31).

### Power analysis

The traditional power analysis for regression-based models is 10 cases per independent variable (33). For the current study, the model contained 5 independent variables and a sample size of N = 56, thereby exceeding the traditional *10–1 rule.* However, while the power of the current model was sufficient to detect population effects, we elected to increase the power of the current model to strengthen our conclusions regarding the generalizability of the results. We did this via the utilization of bootstrap resampling (34).

#### Bootstrapping

With bootstrapping, subsamples are randomly drawn, with replacement, from the original sample. Each subsample is then used to estimate the relationships of the proposed model in the population. This process is repeated many of times, with an N = 5000 often referenced as the minimum number of resamples to execute (34). Research indicates that the bootstrap resampling process produces unbiased population estimates. In the current case, we utilized the Bollen Stine bootstrapping test (35).

The Bollen Stine test operates by comparing the fit of the proposed theoretical model to a model transformed to exactly fit the data. If, after an N = 5000 subsamples, the theorized was not significantly different that the ideal model, it would provide additional evidence of the stability of the model in the population.

## Results

Our analysis began by testing the normality assumptions of ML analysis (31). The results indicated that all items exhibited normality. The zero-order correlations between variables are included in **Table 2**. The zero order correlations exhibited a large positive correlation between the two dimensions of the HLS (*r* = .836; *p* <.001). The sample also demonstrated a positive correlation between income and the HLS dimension of personal and cultural loss.

**Table 2:** Zero order correlations (N = 56).

Variables	1	2	3	4	5
1. Income	1				
2. Number of Children	-.021	1			
3. Mental Health Diagnosis	.102	.092	1		
4. HLS: Government and Institutional Policies	.188	-.038	.099	1	
5. HLS: Personal and Cultural Losses	.297*	.128	.055	.836**	1
6. PPD	-.128	-.113	.042	.255	.145

*p < .05; **p < .001.

Next, we tested the fit of the theorized model. The initial model contained the two dimensions of the HLS are independent predictors of greater PPD symptoms. The initial model also contained a direct path between the HLS dimension of perceptions of oppressive government and institutional policies and PPD symptoms and no direct path between the dimension of HLS: personal and cultural losses. The variables of income, mental health diagnosis, and number of children were used as covariates.

The results of testing the initial model indicated that the model provided good fit to the data (*χ*2 = 13.60; *df* = 10; *p* = .192; RMSEA = .081 [90% CI:.000,.178]; CFI = .949). Moreover, the path from the HLS dimension of oppressive government and institutional policies was statistically significant and positive (*β* = .28, *p* <.05). Based on the heuristics of Cohen (1988), the relationship between HLS and PPD was approaching “moderate.” The overall model accounted for an *R*^2^ = .126 of variance in PPD scores.

We subsequently performed a Bollen-Stine bootstrapping analysis to test the stability of the model. As 5,000 simulated bootstrapped samples, the results indicated that the theorized model was not significantly different than a model that perfectly fits the data (*p* = .144). Such result added evidence of the generalizability of the proposed model to the population.

### Nested models

**Figure 2:**
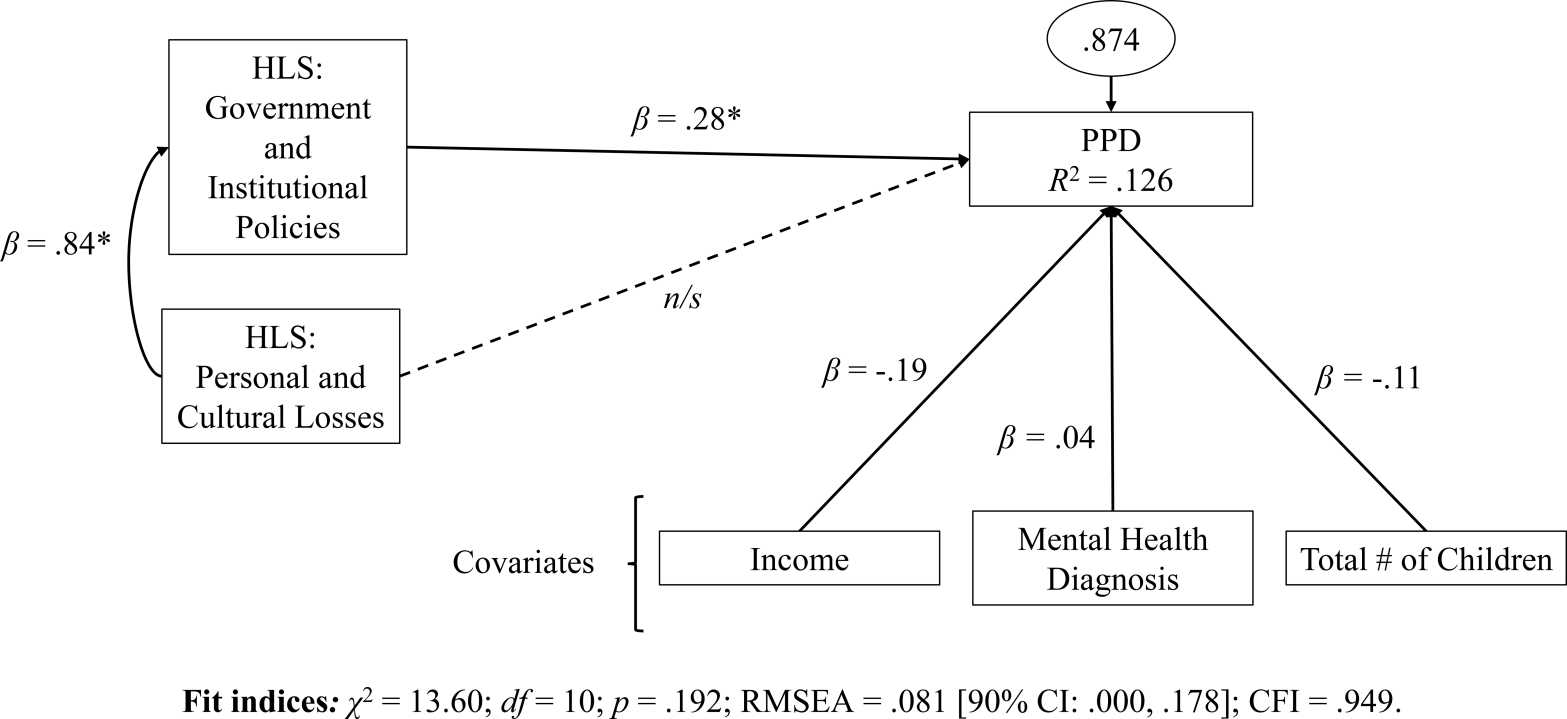
Model of HLS scores as a predictor of post partum depression among a sample of American Indian/Alaska native mothers (standardize values; *N* = 56). Notes. *p < .05

Although the initial model provided good fit to the data, we also evaluated whether adding a path between the second dimension of the HLS scale, the dimension that measures personal and cultural losses, accounted for unique variance in PPD symptoms over and above the HLS dimension of government and institutional policy. The results indicated that the additional path accounted for a Δ*χ*^2^ (1) = .13. Such a change was not a significant improvement in model fit (*p* = .718). Consequently, based on the parsimony principle (31), the additional path was not retained in the final model. **Figure 2** contains all the empirical values of the final model.

## Discussion

The findings from this study provide empirical support for a significant correlation between experiences of HT and PPD. Utilizing the HLS alongside the EPDS allowed for a quantifiable examination of how intergenerational trauma may manifest in contemporary perinatal mental health outcomes. Such a result reinforces the importance of contextualizing maternal mental health within broader historical and sociocultural frameworks. The study results align with and expand upon existing literature that situates HT not only as a collective memory but also as a psychological burden that contributes to adverse health disparities among Indigenous women (36, 37). The findings also align with other research that indicates that the disparities in maternal mental health outcomes that disproportionately affect women of color are related to lifetime stress, which is compounded by stress brought on by racism (38). In addition, the current study aligns with others who have documented that many groups of women who have experienced racism are at an elevated risk of developing PPD symptoms (39). The findings of the current study suggest that the legacies of colonization, forced assimilation, and systemic oppression, in the form of HT, continue to exert a measurable negative influence on maternal mental health today.

### Implications for practice, policy, and advocacy

The current results underscore how PPD symptoms among Indigenous women cannot be fully understood or addressed without acknowledging HT. Our study also suggests the need for a trauma-informed approach to perinatal care that actively recognizes the impact of historical grief and loss.

Insights into the HT-PPD symptoms link can guide the development of healthcare interventions that account for HT, promoting more effective and empathetic care. One key implication of this study is the urgent need to design culturally competent mental health services and screening tools that reflect and honor the lived experiences of diverse populations, particularly Indigenous communities. Standardized tools and interventions often fail to capture the nuanced ways that HT manifests in mental health, leading to underdiagnosis or misdiagnosis of conditions like PPD (40). By integrating culturally specific concepts of wellness, community healing practices, and historical context into screening and care models, mental health providers can build trust, improve diagnostic accuracy, and offer more effective, resonant support. Such approaches not only address individual symptoms but also affirm cultural identity and resilience as central to the healing process.

Another important implication of this study is the need to normalize the impact of trauma by explicitly recognizing HT as a contributing factor to PPD symptoms. Framing mental health struggles within a broader systemic and historical context can help reduce self-blame among affected individuals and families, shifting the narrative from personal weakness to collective experience and resilience. When healthcare providers and systems validate the role of HT, it opens the door for more compassionate, de-stigmatizing conversations around mental health. This recognition not only promotes healing but also empowers individuals and communities to seek support without shame, fostering a more inclusive and trauma-informed approach to maternal care.

An additional implication of this study lies in its potential to drive advocacy and raise awareness about the long-term impacts of HT on maternal mental health. By highlighting the connection between HT and PPD symptoms, this research can inform public discourse, community mobilization, and policy efforts aimed at addressing the root causes of mental health disparities. Increased awareness can empower communities to advocate for systemic changes—such as increased funding for culturally responsive perinatal services, improved access to mental health care, and the incorporation of Indigenous voices in policy development. In this way, the findings contribute not only to academic knowledge but also to broader social and political efforts to promote equity and healing.

The findings in this study also highlight the need to help break intergenerational cycles of trauma by addressing the mental health impacts of HT in mothers. When maternal mental health concerns like PPD are understood and treated within the context of HT, interventions can more effectively target the root causes of distress (Heck et al., 2025). Supporting mothers in healing from trauma not only improves their well-being but also creates a more nurturing and emotionally stable environment for their children. This can reduce the transmission of stress, unresolved grief, and maladaptive coping strategies to future generations, fostering resilience and healthier developmental outcomes (41). In this way, trauma-informed and culturally grounded approaches to maternal mental health can serve as a powerful tool for disrupting cycles of harm and promoting long-term healing across generations. The results of the current study suggest directions for future trauma-informed interventions for Indigenous mothers. The results suggest that trauma informed interventions that target reductions in HT may have the added benefit of mitigating PPD symptoms.

Finally, the evidence linking HT to PPD also holds important implications for policy development. Demonstrating this connection provides a strong justification for policies that aim to reduce health disparities through targeted investments in community-based mental health services and trauma-informed care initiatives. Policymakers can use these findings to advocate for increased funding for culturally responsive perinatal care, support programs that train providers in historical trauma-informed practices and implement screening protocols that capture the unique experiences of marginalized populations. Such policies not only address immediate mental health needs but also work toward long-term systemic change by embedding equity and cultural relevance into the maternal healthcare system.

### Limitations

The current study tested a statistical model that was specified according to the theory that HT across the lifespan experienced prior to birth is a contributing factor to PPD symptoms post birth. Based on this theory, HT was modeled as the independent variable while PPD symptoms were modeled as the dependent variable. However, while this study establishes a correlation, it does prove causation. It is possible that PPD symptoms post birth made the participants more likely to perceive HT. Such a directional relationship would produce the identical correlations identified in the current study. While theory does not support a directional relationship between PPD symptoms and HT, further research using longitudinal designs is necessary to test this assumption and to strengthen the empirical evidence that a greater endorsement of HT is indeed the antecedent to PPD symptoms rather than vice versa.

It should also be noted that given that the current sample included only those that self-identify as Indigenous, generalizations should be made cautiously. The parent population of American Indians is heterogenous, meaning that those who are member of a Federal recognized tribe are not identical to those that self-identify as Indigenous. Future research is needed to better understand how heterogeneity within the Indigenous population might impact the current results. Finally, integrating community-based participatory research and Indigenous methodologies may deepen our understanding of resilience and protective factors that buffer against PPD.

## Conclusion

Understanding the connection between HT and PPD symptoms has critical implications for addressing health disparities and improving maternal mental health outcomes. By shedding light on the relationship between historical trauma and postpartum depression, this research not only contributes to the academic understanding of these phenomena but also informs public health policy and therapeutic interventions tailored to the needs of historically marginalized populations. The findings from this study have the potential to pave the way for transformative approaches to maternal mental health care, rooted in equity and cultural competence.

## Data Availability

The raw data supporting the conclusions of this article will be made available by the authors, without undue reservation.

## References

[1] Heart MY Chase J Elkins J Alt, schul D . Historical trauma among Indigenous peoples of the Americas: concepts, research, and clinical considerations. J Psychoact Drugs. (2011) 43:282–90. doi: 10.1080/02791072.2011.628913 22400458

[2] Praetorius R Maxwell D Alam K . Wearing a happy mask: mother’s expressions of suicidality with postpartum depression. Soc Work Ment Health. (2020) 18(4):429–59. doi: 10.1080/15332985.2020.1769003 41909888

[3] Heck JL . Postpartum depression in American Indian/Alaska Native women: a scoping review. MCN: Am J Maternal/Child Nurs. (2021) 46:6–13. doi: 10.1097/NMC.0000000000000671 33048861

[4] Ko JY Rockhill KM Tong VT Morrow B Farr SL . Trends in postpartum depressive symptoms. MMWR Morb. Mortal. Wkly. Rep. (2017) 66:153–8. doi: 10.15585/mmwr.mm6606a1 28207685 PMC5657855

[5] Whitbeck LB Adams GW Hoyt DR Chen X . Conceptualizing and measuring historical trauma among American Indian people. Am J Community Psychol. (2004) 33(3-4):119–30. doi: 10.1023/b:ajcp.0000027000.77357.31 15212173

[6] Daoud N O'Brien K O'Campo P Harney S Harney E Bebee K . Postpartum depression prevalence and risk factors among Indigenous, non-Indigenous and immigrant women in Canada. Can J Public Health. (2019) 110(4):440–52. doi: 10.17269/s41997-019-00182-8 PMC6964473 30767191

[7] Byrnes L . Perinatal Mood and Anxiety Disorders. J Nurse Pract. (2018) 14(7):507–13. doi: 10.1016/j.nurpra.2018.03.010

[8] Agrawal L Mehendale AM Malhotra R . Risk factors of postpartum depression. Cureus. (2022). doi: 10.7759/cureus.30898 36465774 PMC9711915

[9] Eitenmüller P Köhler S Hirsch O Christiansen H . The impact of prepartum depression and birth experience on postpartum mother-infant bonding: a longitudinal path analysis. Front Psychiatry. (2022) 13:815822. doi: 10.3389/fpsyt.2022.815822 35706472 PMC9189288

[10] Rudzik AEF Robinson-Smith L Tugwell F Ball HL . Relationships between postpartum depression, sleep, and infant feeding in the early postpartum: an exploratory analysis. Front Psychiatry. (2023) 14:1133386. doi: 10.3389/fpsyt.2023.1133386 37032920 PMC10079948

[11] Slomian J Honvo G Emonts P Reginster JY Bruyère O . Consequences of maternal postpartum depression: a systematic review of maternal and infant outcomes. Womens Health (London, England). (2019) 15, 1745506519844044. doi: 10.1177/1745506519844044 31035856 PMC6492376

[12] Yellow Horse Brave Heart M Debruyn L . The American Indian holocaust: healing historical unresolved grief. Am Indian Alaska Nat. Ment Health Res. (1998) 8:56. 9842066

[13] Sotero M . A conceptual model of historical trauma: implications for public health practice and research. J Health Dispar. Pract. (2009) 1:93–108. Available online at: https://www.researchgate.net/publication/228209032.

[14] Gameon JA Skewes MC . A systematic review of trauma interventions in native communities. Am J Community Psychol. (2020) 35(3):223–41. doi: 10.1002/ajcp.12396 31518009 PMC7243818

[15] Gone JP Hartmann WE Pomerville A Wendt DC Klem SH Burrage RL . The impact of historical trauma on health outcomes for Indigenous populations in the USA and Canada: a systematic review. Am Psychol. (2019) 74:20–35. doi: 10.1037/amp0000338 30652897

[16] Derezotes DS . Transforming historical trauma through dialogue. SAGE Publications, Inc. (2014). doi: 10.4135/9781483387536

[17] Evans-Campbell T . Historical trauma in American Indian/Native Alaska communities a multilevel framework for exploring impacts on individuals, families, and communities. J Interpers. Interpers. (2008) 23:316–38. doi: 10.1177/0886260507312290 18245571

[18] John-Henderson NA White EJ Crowder TL . Resilience and health in American Indians and Alaska Natives: a scoping review of the literature. Dev Psychopathol. (2023) 35:2241–52. doi: 10.1017/S0954579423000640 37345444 PMC10739606

[19] Onyewuenyi TL Peterman K Zaritsky E Ritterman Weintraub ML Pettway BL Quesenberry CP . Neighborhood disadvantage, race and ethnicity, and postpartum depression. JAMA Netw Open. (2023) 6:e2342398. doi: 10.1001/jamanetworkopen.2023.42398 37955900 PMC10644210

[20] Dashorst P Mooren TM Kleber RJ de Jong PJ Huntjens RJC . Intergenerational consequences of the Holocaust on offspring mental health: a systematic review of associated factors and mechanisms. Eur J Psychotraumatol. (2019) 10. doi: 10.1080/20008198.2019.1654065 31497262 PMC6720013

[21] Maxwell D Mauldin R Thomas J Holland V . American Indian motherhood and historical trauma: Keetoowah experiences of becoming mothers. Int J Environ Res Public Health. (2022) 19:7088. doi: 10.3390/ijerph19127088 35742333 PMC9222731

[22] Pedhazur EJ . Multiple regression in behavioral research. New York, NY: Harcourt Brace Publishers (1997).

[23] Cox JL Holden JM Sagovsky R . Detection of postnatal depression. Development of the 10-item Edinburgh Postnatal Depression Scale. Br J Psychiatry. (1987) 150:782–6. doi: 10.1192/bjp.150.6.782 3651732

[24] Carroll SR Rodriguez-Lonebear D Martinez A . Indigenous data governance: strategies from United States native nations. Data Sci J. (2019) 18:1–15. doi: 10.5334/dsj-2019-031 34764990 PMC8580324

[25] Whitbeck LB Walls ML Johnson KD Morrisseau AD McDougall CM . Depressed affect and historical loss among north American Indigenous adolescents. Am Indian Alaska Nat. Ment Health Res. (2009) 16:16–41. doi: 10.5820/aian.1603.2009.16 20052631 PMC3235726

[26] Reid KM Taylor MG . Social support, stress, and maternal postpartum depression: a comparison of supportive relationships. Soc Sci Res. (2015) 54:246–62. doi: 10.1016/j.ssresearch.2015.08.009 26463547

[27] Khamidullina Z Marat A Muratbekova S Mustapayeva NM Chingayeva GN Shepetov AM . Postpartum depression epidemiology, risk factors, diagnosis, and management: an appraisal of the current knowledge and future perspectives. J Clin Med. (2025) 14:2418. doi: 10.3390/jcm14072418 40217868 PMC11989329

[28] Amer SA Zaitoun NA Abdelsalam HA Abbas A Ramadan MS Ayal HM . Exploring predictors and prevalence of postpartum depression among mothers: Multinational study. BMC Public Health. (2024) 24:1308. doi: 10.1186/s12889-024-18502-0 38745303 PMC11092128

[29] Pocock SJ Assmann SE Enos LE Kasten LE . Subgroup analysis, covariate adjustment and baseline comparisons in clinical trial reporting: current practice and problems. Stat Med. (2002) 21:2917–30. doi: 10.1002/sim.1296 12325108

[30] Arbuckle JL . AMOS 19 [Computer software]. Chicago, IL: SPSS (2010).

[31] Kline RB . Principles and practice of structural equation modeling. New York, NY: Guilford Press (2016).

[32] Browne MW Cudeck R . Alternative ways of assessing model fit. In: Bollen KA Long JS , editors.Testing structural equation models. Sage Publications, Newbury Park, CA (1993). p. 136–62.

[33] Field A . Discovering statistics using IBM SPSS Statistics. London: Sage (2018).

[34] Hair JF Hult GTM Ringle CM Sarstedt M . A primer on partial least squares structural equation modeling (PLS-SEM). Thousand Oaks, CA: SAGE Publications (2014).

[35] Bollen KA Stine RA . Bootstrapping goodness-of-fit measures in structural equation models. In: Bollen KA Long JS , editors.Testing structural equation models. Sage, Newbury Park, CA (1993). p. 111–35.

[36] Ehrenpreis JE Ehrenpreis ED . A historical perspective of healthcare disparity and infectious disease in the Native American population. Am J Med Sci. (2022) 363:288–94. doi: 10.1016/j.amjms.2022.01.005 35085528 PMC8785365

[37] Walters KL Simoni JM . Reconceptualizing native women's health: an "indigenist" stress-coping model. Am J Public Health. (2002) 92:520–4. doi: 10.2105/ajph.92.4.520 11919043 PMC1447108

[38] Dominguez TP Dunkel-Schetter C Glynn LM Hobel C Sandman CA . Racial differences in birth outcomes: the role of general, pregnancy, and racism stress. Health Psychol. (2008) 27:194–203. doi: 10.1037/0278-6133.27.2.194 18377138 PMC2868586

[39] Weeks F Zapata J Rohan A Green T . Are experiences of racial discrimination associated with postpartum depressive symptoms? a multistate analysis of pregnancy risk assessment monitoring system data. J Womens Health. (2022). doi: 10.1089/jwh.2021.0426 34967671 PMC10941332

[40] Heck JL . Screening for postpartum depression in American Indian/AlaskaNative women: a comparison of two instruments. Am Indian Alsk Nat. Ment Health Res. (2018) 25:74–102. doi: 10.5820/aian.2502.2018.74 29889949

[41] Reddy S Patel N Saxon M Amin N Biviji R . Innovations in U.S. health care delivery to reduce disparities in maternal mortality among African American and American Indian/Alaskan Native women. J Patient Cent Res Rev. (2021) 8:140–5. doi: 10.17294/2330-0698.1793 33898647 PMC8060040

